# Neurotropic Lineage III Strains of Listeria monocytogenes Disseminate to the Brain without Reaching High Titer in the Blood

**DOI:** 10.1128/mSphere.00871-20

**Published:** 2020-09-16

**Authors:** Taylor E. Senay, Jessica L. Ferrell, Filip G. Garrett, Taylor M. Albrecht, Jooyoung Cho, Katie L. Alexander, Tanya Myers-Morales, Olivia F. Grothaus, Sarah E. F. D’Orazio

**Affiliations:** a Department of Microbiology, Immunology & Molecular Genetics, University of Kentucky College of Medicine, Lexington, Kentucky, USA; b Department of Pathology and Laboratory Medicine, University of Kentucky College of Medicine, Lexington, Kentucky, USA; University of Iowa

**Keywords:** *Listeria monocytogenes*, foodborne infection, mouse models

## Abstract

Listeria monocytogenes is thought to colonize the brain using one of three mechanisms: direct invasion of the blood-brain barrier, transportation across the barrier by infected monocytes, and axonal migration to the brain stem. The first two pathways seem to occur following unrestricted bacterial growth in the blood and thus have been linked to immunocompromise. In contrast, cell-to-cell spread within nerves is thought to be mediated by a particular subset of neurotropic L. monocytogenes strains. In this study, we used a mouse model of foodborne transmission to evaluate the neurotropism of several L. monocytogenes isolates. Two strains preferentially colonized the brain stems of BALB/cByJ mice 5 days postinfection and were not detectable in blood at that time point. In contrast, infection with other strains resulted in robust systemic infection of the viscera but no dissemination to the brain. Both neurotropic strains (L2010-2198, a human rhombencephalitis isolate, and UKVDL9, a sheep brain isolate) typed as phylogenetic lineage III, the least characterized group of L. monocytogenes. Neither of these strains encodes InlF, an internalin-like protein that was recently shown to promote invasion of the blood-brain barrier. Acute neurologic deficits were observed in mice infected with the neurotropic strains, and milder symptoms persisted for up to 16 days in some animals. These results demonstrate that neurotropic L. monocytogenes strains are not restricted to any one particular lineage and suggest that the foodborne mouse model of listeriosis can be used to investigate the pathogenic mechanisms that allow L. monocytogenes to invade the brain stem.

**IMPORTANCE** Progress in understanding the two naturally occurring central nervous system (CNS) manifestations of listeriosis (meningitis/meningoencephalitis and rhombencephalitis) has been limited by the lack of small animal models that can readily distinguish between these distinct infections. We report here that certain neurotropic strains of Listeria monocytogenes can spread to the brains of young otherwise healthy mice and cause neurological deficits without causing a fatal bacteremia. The novel strains described here fall within phylogenetic lineage III, a small collection of L. monocytogenes isolates that have not been well characterized to date. The animal model reported here mimics many features of human rhombencephalitis and will be useful for studying the mechanisms that allow L. monocytogenes to disseminate to the brain stem following natural foodborne transmission.

## Abstract (precis)

Progress in understanding the two naturally occurring central nervous system (CNS) manifestations of listeriosis (meningitis/meningoencephalitis and rhombencephalitis) has been limited by the lack of small animal models that can readily distinguish between these distinct infections. We report here that certain neurotropic strains of Listeria monocytogenes can spread to the brains of young otherwise healthy mice and cause neurological deficits without causing a fatal bacteremia. The novel strains described here fall within phylogenetic lineage III, a small collection of L. monocytogenes isolates that have not been well characterized to date. The animal model reported here mimics many features of human rhombencephalitis and will be useful for studying the mechanisms that allow L. monocytogenes to disseminate to the brain stem following natural foodborne transmission.

## INTRODUCTION

The facultative intracellular bacterium Listeria monocytogenes causes two different types of central nervous system infections: diffuse meningoencephalitis and a focal brain stem infection (rhombencephalitis). L. monocytogenes is a common cause of bacterial meningitis in patients with some degree of immune compromise, such as neonates and the elderly (1–3). Rhombencephalitis is a less common manifestation of listeriosis (4), but interestingly, most of these infections occur in otherwise healthy individuals (5–7). This suggests that brain stem infections are likely caused by specific neurotropic strains of L. monocytogenes rather than by a generalized defect in immunity. Symptoms of rhombencephalitis usually appear in two phases: nonspecific headache and malaise, and then sudden onset of late-stage asymmetric cranial nerve deficits, hemiparesis, or balance and movement difficulties (8–10). Rhombencephalitis is the most common presentation of listeriosis in ruminants, and it has been suggested that cattle or sheep may be a natural reservoir for neurotropic strains that cause brain stem infections in humans (11–13).

Three mechanisms have been proposed to explain how L. monocytogenes disseminates to the brain: direct invasion of the blood-brain barrier, transport inside or attached to a migratory immune cell, and axonal migration. Recent work showed that the L. monocytogenes surface protein InlF is important for optimal invasion of brain endothelial cells *in vitro* and colonization of the murine brain following intravenous inoculation (14). Systemic listeriosis induces proinflammatory cytokine expression in the brain that precedes an influx of Ly6C^hi^ monocytes, and L. monocytogenes has been found associated with those infiltrating cells (15–18). The best evidence to suggest that axonal migration of L. monocytogenes results in brain stem infection comes from the study of naturally infected sheep where bacteria were observed within myelinated axons of the trigeminal nerve (19). The Oevermann group has also shown that L. monocytogenes can move in an ActA-dependent manner within the axon-like processes of cultured bovine brain cells (20).

Hematogenous spread of L. monocytogenes can be modeled in mice by intravenously injecting large doses of reference strains such EGD or 10403s (14, 15). Early studies indicated that a prolonged bacteremia with 10^2^ to 10^3^ CFU/ml of blood was needed for brain colonization (21, 22). Other routes of transmission have been attempted, including subcutaneous injection (23) and direct inoculation of the cisterna magna (24). However, in these studies, the doses used were either at or significantly above the 50% lethal dose (LD_50_), and histopathology was indicative of meningitis. The only published description of a mouse model of rhombencephalitis required daily oral doses for 7 to 10 days and resulted in late-stage neurological deficits and brain stem lesions in only 25% of the infected animals (25).

L. monocytogenes strains are divided into four evolutionary lineages, with the majority of isolates characterized to date belonging to lineages I and II (26). Lineage II strains are commonly isolated from both food products and patients with sporadic infections, but most of the defined listeriosis outbreaks have been caused by lineage I strains (26). A subset of lineage I strains were hypervirulent in mouse models (27, 28), and sequence type 1 (ST1) strains, in particular, were strongly associated with rhombencephalitis in ruminants (29). Much less is known about lineage III and lineage IV due to the smaller number of strains isolated and characterized. Lineage III strains appear to be overrepresented among veterinary isolates and less frequently found in food products, perhaps due to their reduced ability to replicate at refrigerated temperatures (30–32). The virulence potential of lineage III isolates is not yet clear, with some reports describing strains being isolated from both animals and humans with clinical disease, but other studies suggesting that the strains may lack key virulence factors (33).

We previously described a mouse model of listeriosis that closely mimics key features of human disease, including foodborne transmission, a discrete phase during which L. monocytogenes organisms are present only in the gastrointestinal tract, various degrees of host susceptibility in different mouse strains, and late-stage spread to the brain (34–36). In the course of these studies, we noticed that some mouse-adapted (InlA^m^-expressing) strains of L. monocytogenes reached the brain late in the infection cycle but were not detected in the blood. This led us to hypothesize that there were neurotropic strains of L. monocytogenes that could invade the nervous system and colonize the brain using a mechanism that did not involve hematogenous spread. In this study, we tested a small collection of L. monocytogenes isolates in the foodborne mouse model to see if any strain could reproduce the features of rhombencephalitis. We identified two new clinical isolates that colonized the murine brain without reaching high titer in the blood, and surprisingly, both of the isolates typed as lineage III strains. Here, we describe a mouse model of infection using these novel strains that results in both acute and lingering neurological deficits and has a low rate of mortality in otherwise healthy animals.

## RESULTS

### Neurotropic strains of L. monocytogenes disseminate to the brain.

We previously reported that foodborne transmission of mouse-adapted (InlA^m^-expressing) L. monocytogenes resulted in nonlethal colonization of the brain in mice (34). This suggested that the mouse model of foodborne listeriosis might be useful for studying how L. monocytogenes disseminates to the brain to cause rhombencephalitis, an infection that typically presents in otherwise healthy patients as cranial nerve deficits with a low mortality rate (5, 6, 9). We postulated that strains isolated from naturally occurring brain stem infections would be neurotropic and would invade neural tissue and spread to the brain, while other virulent strains of L. monocytogenes would replicate primarily in the intestines, spleen, and liver and spread to the brain only if they reached high titer in the blood. To test this, we compared the ability of L. monocytogenes EGDe, the mouse-adapted variant EGDe-InlA^m^, and other human and veterinary isolates to colonize the brains of mice following foodborne transmission. The origins of the strains used in this study are presented in Table 1.

**TABLE 1:** Listeria monocytogenes strains used in this study

Strain	Origin	Reference or source	MLST^*a*^ (*abcZ bglA cat dapE dat ldh lhkA*)	ST	Lineage
EGDe	Rabbit	38	*6 5 6 20 1 4 1*	ST35	II
EGDe-InlA^m^	EGDe derivative	38	*6 5 6 20 1 4 1*	ST35	II
UKVDL4	Cow liver	UK VDL^*b*^	*204 105 194 24 151 145 161*	ST1069	III
UKVDL7	Horse liver	UK VDL	*189 159 99 225 32 355 150*	ST1140	III
UKVDL9	Sheep brain	UK VDL	*116 33 204 253 34 388 28*	ST1194	III
L2010-2198	Human (rhombencephalitis)	CDC^*c*^	*58 60 68 48 41 349 44*	ST1590	III

a Allele numbers provided by the Institut Pasteur MLST Database.

b University of Kentucky Veterinary Diagnostic Laboratory.

c Centers for Disease Control and Prevention, National *Listeria* Reference Laboratory.

**FIG 1:**
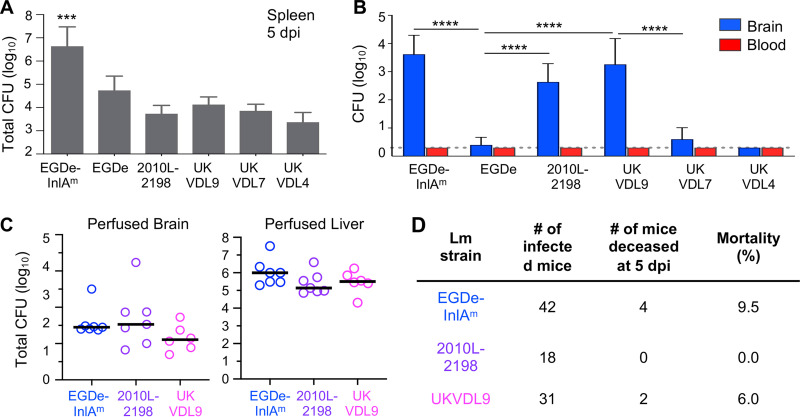
Neurotropic L. monocytogenes disseminates to the brain without reaching detectable levels in the blood. Female BALB/cByJ mice were fed 10^9^ CFU of the indicated L. monocytogenes (Lm) strain. (A and B) Total CFU in homogenates of whole brain or spleen and the total CFU per milliliter in whole blood were determined 5 days postinfection (dpi). The dashed line indicates the limit of detection. Mean values and standard deviations (SD) for pooled data (5 to 7 mice per group) from two separate experiments are shown. Data in panel A were analyzed by one-way analysis of variance (ANOVA) with Tukey’s multiple-comparison test; data in panel B were analyzed by two-way ANOVA. ***, *P < *0.001; ****, *P < *0.0001. (C) Five days postinfection, cardiac perfusion was performed, and the total number of CFU in homogenates of whole brain or liver was determined. Pooled data from two separate experiments are shown; horizontal lines indicate median values. (D) Overall mortality rate at 5 dpi for mice fed neurotropic L. monocytogenes. Pooled data from multiple experiments are shown.

Groups of female BALB/cByJ mice were fed ∼1 × 10^9^ CFU of each of the six strains listed in Table 1, and the total numbers of CFU found in the spleen and brain were determined 5 days later. This time point was chosen because it was the earliest time at which L. monocytogenes was detected in the brain in our previous studies using this foodborne model of infection (34, 37). As expected, the mouse-adapted strain EGDe-InlA^m^ colonized the spleen significantly better than the other five strains (Fig. 1A). However, at least 10^3^ CFU were present in the spleen for all strains tested, indicating that BALB/cByJ mice were also susceptible to infection with the strains that were not mouse adapted.

Although all of the strains fed to mice caused systemic infection, only three strains were found at high titer in whole-brain homogenates: EGDe-InlA^m^, the murinized form of L. monocytogenes EGDe; L2010-2198, a human rhombencephalitis isolate; and UKVDL9, a sheep brain isolate (Fig. 1B). In contrast, wild-type L. monocytogenes EGDe and two other veterinary strains (UKVDL7 and UKVDL4) were either at or below the limit of detection in the brain 5 days postinfection. This result suggested that EGDe-InlA^m^, L2010-2198, and UKVDL9 might be neurotropic and could more efficiently colonize the brain than other strains of L. monocytogenes. None of the tested strains were detectable in the blood at 5 days postinfection (Fig. 1B), suggesting that concurrent high-titer bacteremia was not required for invasion of the brain and that the mechanism involved might not be hematogenous spread.

To further confirm that the L. monocytogenes organisms detected were in the brain parenchyma and not simply circulating within blood vessels of the brain, we performed cardiac perfusion to remove blood prior to organ harvest. As shown in Fig. 1C, L. monocytogenes were readily recovered from the perfused brains and livers of all infected mice. For mice fed either L. monocytogenes EGDe-InlA^m^ or L. monocytogenes L2010-2198, approximately 10^3^ CFU were present in perfused brains (Fig. 1C), similar to the bacterial burdens observed in whole-brain homogenate (Fig. 1B). On average, about 10-fold-fewer L. monocytogenes were recovered from the perfused brains of mice infected with L. monocytogenes UKVDL9. Thus, for all three strains, most of the detectable bacteria were found in the brain parenchyma, supporting the hypothesis that hematogenous spread due to high-titer bacteremia was not the primary mechanism of brain colonization.

In the course of these experiments, a few mice succumbed to the infection and were excluded from the analysis. The overall mortality at 5 days postinfection for all animals used in this study is shown in Fig. 1D. L. monocytogenes EGDe-InlA^m^ and L. monocytogenes UKVDL9 both had low mortality rates, at 9.5% and 6%, respectively. L. monocytogenes L2010-2198 caused no fatalities at 5 days postinfection (Fig. 1D). Together, these results indicated that in the majority of animals, colonization of the brain was not acutely lethal.

### Neurotropic strains of L. monocytogenes belong to lineage III.

Multilocus sequence typing (MLST) was performed to facilitate phylogenetic analysis of the neurotropic L. monocytogenes. Both the control strain EGDe and the mouse-adapted variant EGDe-InlA^m^ typed as ST35, the expected sequence type in lineage II (Table 1). Surprisingly, all four of the remaining strains typed as belonging to lineage III. The three veterinary isolates (UKVDL4, UKVDL7, and UKVDL9) each had individual allelic profiles but matched sequence types (ST1069, ST1140, and ST1194, respectively) that were already present in the Institut Pasteur *Listeria* MLST database. In contrast, the human isolate L2010-2198 had a new allelic profile (*abcZ58 bglA60 cat68 dapE48 dat41 ldh349 lhkA44*) that was given the *de novo* designation ST1590 (Table 1). Thus, the two newly identified clinical isolates that disseminated to the brain in the foodborne mouse model (UKVDL9 and L2010-2198) were both lineage III strains.

The third strain that demonstrated some neurotropism was the mouse-adapted variant of L. monocytogenes EGDe. The only known modifications in this strain compared to wild-type EGDe are two amino acid substitutions in internalin A (S192N and Y369S), which increase affinity for murine E-cadherin (38) but also allow binding to N-cadherin (39). N-cadherin is highly expressed in neural tissue (40), so it is possible that this modification could allow invasion of cranial nerves within the gastrointestinal tract. The tyrosine-to-serine substitution, in particular, could readily occur with a single base pair mutation (TAT to TCT or TAC to TCC), raising the possibility that these amino acid substitutions might naturally occur in L. monocytogenes. We examined the predicted amino acid sequences for *inlA* encoded by 531 publicly available L. monocytogenes strains as well as the six strains used in this study but did not find any naturally occurring S192N or Y369S substitutions (Data Set S1). The collection included 146 environmental isolates, 145 strains from food products, 133 human clinical isolates (16 with known central nervous system [CNS] involvement), 97 veterinary isolates (9 with CNS origin), and 16 strains of uncertain origin. These results suggested that the InlA^m^ modification that allows efficient oral transmission in mice and could possibly promote neurotropism is not commonly found in other L. monocytogenes isolates.

10.1128/mSphere.00871-20.4 DATA SET S1 Excel file of InlA sequences. Download Data Set S1, XLSX file, 0.2 MB. Copyright © 2020 Senay et al. 2020 Senay et al. This content is distributed under the terms of the Creative Commons Attribution 4.0 International license.

### InlF is not required for lineage III strains to invade the brain.

**FIG 2:**
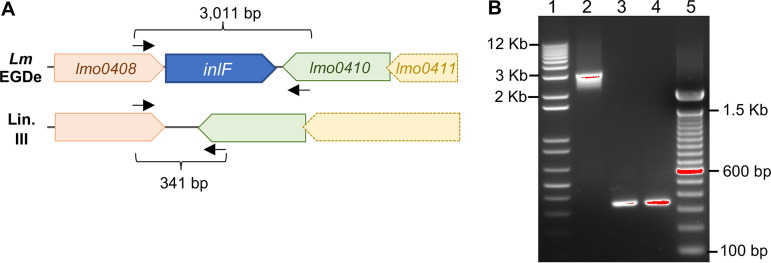
Neurotropic strains UKVDL9 and 2010L-2198 lack *inlF*. (A) In L. monocytogenes EGDe, *inlF* is positioned between *lmo0408* and *lmo0410* (GenBank accession number NC_003210.1). Open reading frames (ORFs) that are nearly identical to *lmo0408* and *lmo0410* exist within lineage III strains, including M7, HCC23, SLCC2376, and L99 (GenBank accession numbers NC_017537.1, NC_011660.1, NC_018590.1, and NC_017529); however, these strains lack *inlF* and have only a 70-bp intergenic sequence. Primers used to amplify this region are indicated by arrows, and the predicted sizes of the amplicons are noted. (B) PCR amplification of the *lmo0408-inlF-lmo0410* region from genomic DNA. Lane 1, 1 Kb Plus DNA ladder; lane 2, L. monocytogenes EGDe-InlA^m^ amplicon; lane 3, L. monocytogenes L2010-2198 amplicon; lane 4, L. monocytogenes UKVDL9 amplicon; lane 5, 100 bp DNA ladder.

Ghosh et al. found that a member of the internalin family of proteins (InlF) was important for crossing the blood-brain barrier during systemic L. monocytogenes infection (14). In that study, the bacteria were administered intravenously, and at least 10^2^ CFU/ml were found in the blood of all infected animals 3 days postinfection, when brain colonization occurred. InlF is encoded within the gene cluster *lmo0408* to *lmo0411* in reference strain L. monocytogenes EGDe (Fig. 2A). Lineage III strains with publicly available whole-genome sequences contain this gene cluster but lack *inlF*. To find out if neurotropic strains UKVDL9 and L2010-2198 contained *inlF*, we designed primers to amplify this region of the chromosome (Fig. 2A). As expected, the full-length fragment was amplified using genomic DNA from EGDe-InlA^m^ (Fig. 2B). However, a smaller fragment lacking *inlF* was detected in both lineage III neurotropic strains. These results are consistent with a previously published study which found that *inlF* could not be amplified from eight additional lineage III strains (41). In addition, draft genome sequences for UKVDL9 and L2010-2198 did not contain any regions with high similarity to *inlF* (data not shown). Thus, the neurotropism of L2010-2198 and UKVDL9 cannot be explained by expression of InlF, suggesting that an alternate mechanism of brain invasion may be involved.

### Neurotropic L. monocytogenes strains preferentially colonize the brain stem.

Focal brain stem infection (rhombencephalitis) is thought to be caused by invasion of nerves and subsequent axonal migration of L. monocytogenes to the base of the brain (42). In contrast, hematogenous spread of L. monocytogenes via invasion of the blood-brain barrier likely leads to a more diffuse infection of the brain. Therefore, identifying the site of initial colonization in the brain can provide insight into the dissemination mechanism used by L. monocytogenes. We predicted that dissemination via axonal migration would result in colonization primarily within the brain stem, while hematogenous spread would result in more uniform distribution of bacteria in the whole brain.

**FIG 3:**
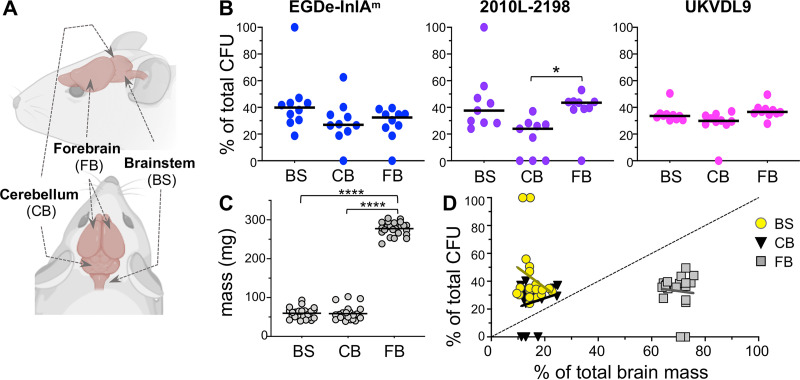
Neurotropic L. monocytogenes preferentially localize to the brain stem. Female BALB/cByJ mice were fed ∼3 × 10^9^ CFU of the indicated L. monocytogenes strain. (A) Brains were harvested 5 dpi and sectioned into brain stem (BS), cerebellum (CB), and forebrain (FB). Image adapted from stock template at Biorender.com; see also Fig. S1A for representative photos of sectioned tissues. (B) Symbols indicate the percentages of CFU found in brain sections of individual mice. Pooled data from at least three separate experiments are shown in each graph; horizontal lines indicate median values. Cardiac perfusion was performed in some experiments (see Fig. S2B). (C) Symbols represent the weights of each tissue section (pooled from all infected brains); horizontal lines indicate mean values. Data in panels B and C were analyzed by one-way ANOVA with Tukey’s multiple-comparison tests. *, *P < *0.5; ****, *P < *0.0001. (D) The percentage of L. monocytogenes per tissue section (same data as shown in panel B, pooled from all strains) is plotted as a function of percentage of the total brain mass. The dashed line represents a theoretical 1:1 ratio of CFU burden and tissue mass (*y* = *x*). Solid lines indicate best linear regressions for each brain section. Regression equations were strongly preferred over *y* = *x* when Akaike information criteria (AICc) were compared.

To test this, we infected groups of BALB/cByJ mice with each of the neurotropic strains, harvested the brains 5 days postinfection, and sectioned each brain into three parts: brain stem (including the medulla and pons), cerebellum, and forebrain (Fig. 3A). As shown in Fig. 3B, two mice had focal brain infections; one mouse that was fed strain EGDe-InlA^m^ and one that was fed strain L2010-2198. In both cases, 100% of the CFU recovered from those mice were found in the brain stem. For the remainder of the mice, L. monocytogenes was recovered from all three brain sections (Fig. 3B). Although there was a trend toward a higher CFU burden in the brain stem than the cerebellum for both L. monocytogenes EGDe-InlA^m^ and L. monocytogenes L2010-2198, no significant differences were found between the mean values for total CFU in the three brain sections.

10.1128/mSphere.00871-20.1 FIG S1 Supplemental information to support data shown in Fig. 3. (A) Typical appearance of perfused and unperfused brains, sectioned into forebrain, cerebellum, and brain stem (left to right). (B) The data set shown in Fig. 3B is further differentiated by perfusion status with symbol shape indicating mice that were fully perfused (triangle), partially perfused (square), or unperfused (circle). BS, brain stem; CB, cerebellum; FB, forebrain. (C) The data set shown combined in Fig. 3D is separated by L. monocytogenes strain here. Blue circles, L. monocytogenes EGDe-InlA^m^; purple squares, L. monocytogenes L2010-2198; pink triangles, L. monocytogenes UKVDL9. Download FIG S1, PDF file, 2.0 MB. Copyright © 2020 Senay et al. 2020 Senay et al. This content is distributed under the terms of the Creative Commons Attribution 4.0 International license.

10.1128/mSphere.00871-20.2 FIG S2 Supplemental information to support data shown in Fig. 6. The rubric used to assign neurological deficit scores (0, 1, 2, or 3) is shown. Daily assessments of mice were performed by two independent investigators who were blinded to the study groups. Each investigator recorded their own observations at separate times during the day. At the completion of the study, the results were unblinded and an average daily value was calculated for each mouse. Download FIG S2, PDF file, 0.02 MB. Copyright © 2020 Senay et al. 2020 Senay et al. This content is distributed under the terms of the Creative Commons Attribution 4.0 International license.

The forebrains had an average mass that was nearly five times greater than that of either the brain stem or cerebellum (Fig. 3C). If dissemination to the brain occurred diffusely, one would expect the percentage of bacteria in a given section to be proportional to total mass of that tissue. However, for all three L. monocytogenes strains, the percentage of CFU in the forebrains was much lower than the expected ratio, with all values below the theoretical *y* = *x* line (Fig. 3D and Fig. S1). For the cerebellums, values above and below the *y* = *x* line were observed, with the average falling just above the theoretical 1:1 ratio. However, brain stems had a higher CFU-to-mass ratio, with all values lying above the hypothetical *y* = *x* line (Fig. 3D), suggesting that L. monocytogenes preferentially colonized this part of the brain. Regression analysis resulted in *R*^2^ values less than 0.06 for all tissue sections, indicating that the data were nonlinear (Fig. 3D). Together, these results were consistent with a model in which L. monocytogenes initially invaded the brain stem and then further disseminated to the cerebellum and eventually to the forebrain in most animals.

### Foodborne infection with strain L2010-2198 causes focal meningoencephalitis.

**FIG 4:**
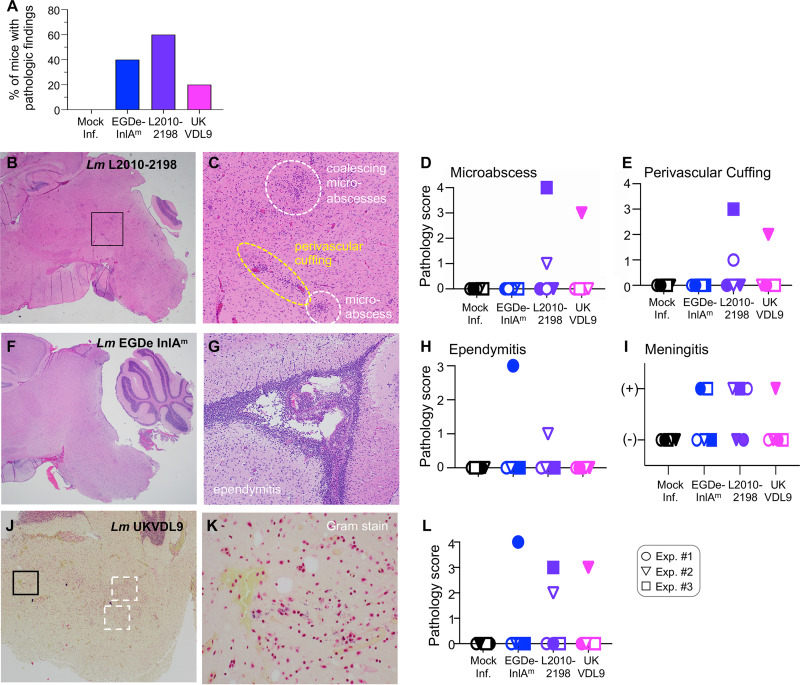
Histopathological findings in the brain 6 days postinfection. BALB/cByJ mice (1 or 2/group) were fed the indicated strain of L. monocytogenes (actual doses ranged from 5 × 10^8^ to 1 × 10^9^ CFU) or mock infected. Brains were sectioned and stained with hematoxylin and eosin (H&E) at 6 dpi; blinded slides were evaluated by a neuropathologist, and the severity of each pathological finding was scored. Pooled data from three separate experiments (total of 5/group) are shown. Filled and unfilled circles (experiment 1), triangles (experiment 2), and squares (experiment 3) indicate values for individual mice. (A) Percentage of mice in each group with abnormal histological findings. (B and C) Representative images for rhombencephalitis from an L. monocytogenes L2010-2198-infected mouse depict a 20× view of the brain stem (B) and a 100× view of the boxed area (C). The extent of microabscess formation (D) and perivascular cuffing (E) observed in each mouse is shown. (F and G) Severe ependymitis in the lateral ventricle of an L. monocytogenes EGDe-InlA^m -^infected mouse at ×100 (G) and a ×20 view of the brain stem of the same mouse (F). The incidence of ependymitis observed in each group of mice is shown in panel H. (I) Focal meningitis in the brain stem was noted as either present or absent in each mouse. (J) Representative image of Gram-stained sections from mice with a focal brain stem infection at ×40. Boxes indicate areas where bacteria were observed at higher magnification. (K) View of the black-boxed area in panel J at ×600. (L) The relative quantity of bacteria in each brain section was scored.

To further confirm brain stem localization of the bacteria and to identify pathological lesions in the brain, we performed a histological analysis on another group of animals. Brains were bisected medially, and thin sections from each hemisphere were prepared 6 days postinfection, a time point when L. monocytogenes was presumed to have been in the brain for at least 24 h. As shown in Fig. 4A, pathological findings were noted at this early time point in each of the groups infected with neurotropic strains; no abnormalities were noted in the brains of mock-infected mice. In mice fed either strain UKVDL9 or L2010-2198, lesions were found primarily in the medulla and pons, with extension into the midbrain in a few animals, a finding consistent with the CFU determinations. Microabscesses and perivascular cuffing were the predominant findings in mice infected with these two neurotropic strains (Fig. 4B to E); meningitis was also noted in all of these animals (Fig. 4I). In contrast, mice infected with strain EGDe-InlA^m^ displayed only meningitis or ependymitis (inflammation of the ventricles, indicative of spread from the spinal fluid), with no infiltrates in the brain stem parenchyma noted (Fig. 4F to H). Gram stains of the same tissue sections indicated the presence of Gram-positive bacilli that colocalized with the abnormal lesions in the brain (Fig. 4J to L). No bacteria were observed in the brains of mice that lacked pathology findings. The lower incidence of brain infection observed in this experiment compared to the previous determination of live replicating CFU in whole brains (Fig. 3) likely reflects the limitation of examining only a portion of the tissue by microscopic analysis. Together, these findings confirmed that strains UKVDL9 and L2010-2198 caused a focal encephalitis in the brain stems of mice following foodborne transmission.

### Neurotropic L. monocytogenes strains cause both acute and persistent neurological deficits.

Rhombencephalitis typically presents with the sudden onset of neurological symptoms, including cranial nerve palsies and cerebellar ataxia in humans and cranial nerve palsies and repetitive circling in ruminants (9, 43, 44). These deficits may be alleviated when the infection is cleared, but in many cases, they persist past the period of active infection and can result in lifelong neurological impairment (4, 9). Thus, to closely mimic human rhombencephalitis, a mouse model should cause a similar pattern of widespread acute neurological deficits with lingering persistent defects observed in at least some animals.

**FIG 5:**
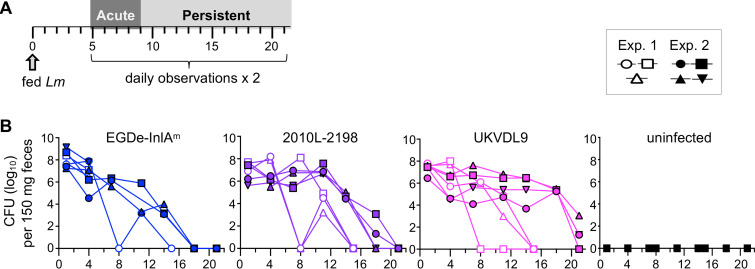
Bacterial burdens during the 3-week observational study. (A) Timeline for the experimental design. Groups of female BALB/cByJ mice (3 or 4 per group) were infected with 1 × 10^9^ CFU of the indicated strain; a control group was mock infected by being fed uncontaminated food. Starting at 5 dpi, mice were subjected to testing and observation for neurological deficits by two independent investigators. (B) Fecal shedding of L. monocytogenes CFU as determined by plating on BHI/L+G plates. Lines indicate patterns for individual mice, with animals from experiment (Exp.) 1 represented by open symbols and mice from experiment 2 by filled symbols.

To test this, groups of female BALB/cByJ mice were infected with either L. monocytogenes EGDe-InlA^m^, UKVDL9, or L2010-2198 in two separate trials (Fig. 5A). A control group of mice mock infected with uncontaminated food was also observed for 3 weeks. Daily assessments for neurological impairment in each mouse were scored according to a rubric (Fig. S2) by two blinded, independent investigators. At the end of the observation period, the study was unblinded and mean values for daily ledge test, gait impairment, and circling disease scores were determined. No observations of ptosis (eyelid drooping) or whisker paralysis were noted throughout the study period (data not shown). Severe symptoms were observed from days 5 to 9 postinfection in both experiments, and this time period was thereafter referred to as the acute phase (Fig. 5A). During the remaining 12 days of the observation period, only low-level deficits were noted, so this period was designated the persistent phase.

Uniform exposure to L. monocytogenes in each mouse was verified by assessing the amount of CFU shed in feces 3 h postinfection, and intestinal infection was monitored every few days after that for the remainder of the observation period. Fecal counts persisted in several mice but generally trended downward (Fig. 5B). These data were consistent with our prior studies, which showed fecal shedding of orally acquired L. monocytogenes long after the systemic infection was cleared from the spleen or liver (34, 45). Mice in the uninfected control group had no detectable L. monocytogenes in feces (Fig. 5B).

**FIG 6:**
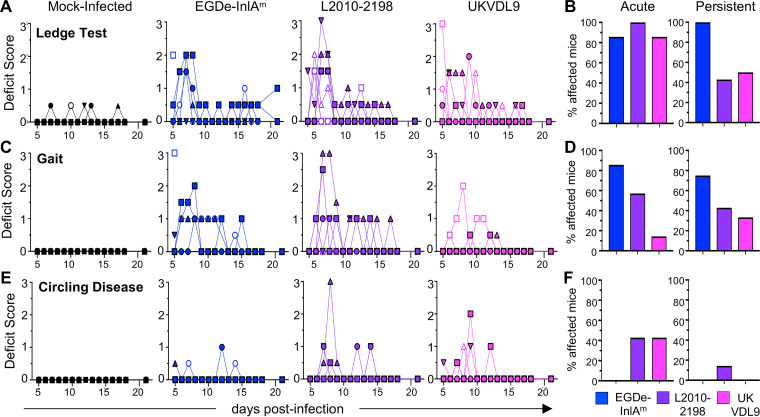
Infection with neurotropic L. monocytogenes results in both acute and persistent neurological deficits. Groups of mice were infected and observed as described in the legend to Fig. 4A. Deficit scores for the ledge test (A), gait (C), and circling disease (E) were recorded daily by two different investigators; mean values for individual mice (*n *=* *7) are shown. Animals from experiment 1 are represented by open symbols and mice from experiment 2 by filled symbols. The rubric used for assigning deficit scores for each test is provided in Fig. S2. (B, D, and F) The overall percentage of mice that displayed either acute or persistent neurological deficits is indicated for each L. monocytogenes strain. During the acute phase, mice were considered affected if they received a score of ≥1 at any point between 5 and 9 dpi; during the persistent phase, mice were classified as affected if they received a score of ≥0.5 for at least two consecutive days.

Cerebellar ataxia was assessed by performing a ledge test (Fig. 6A), a direct measure of coordination during which the mouse must walk along the edge of a cage and lower itself back into the cage using both its paws and hind legs. The majority of the infected mice displayed acute deficits in the ledge test, regardless of the strain used for infection (Fig. 6B). However, the highest median peak deficit scores occurred in mice infected with strain L2010-2198 (Fig. 6A). During the persistent phase, all mice infected with EGDe-InlA^m^ and nearly half of the mice infected with the other two strains also had either lingering or late-appearing deficits (Fig. 6B). It should be noted that a few of the mock-infected mice were also given nonzero results during the observation period (Fig. 6A). However, these were sporadic observations (i.e., never seen in the same animal on more than 1 day and never observed by more than one investigator on the same day). Acute deficits in the infected mice were distinguished by their increased severity, while persistent defects as quantified in Fig. 6B were distinguished by their recurrent observation in the same animal on more than one consecutive day.

Gait impairment was assessed by noting any limp or tremor in the front paws or hind legs or any changes in posture that resulted in dragging of the abdomen. Again, the most severe acute gait deficits were observed in mice infected with strain L2010-2198 (Fig. 6C). Mice fed EGDe-InlA^m^ had the greatest percentage of affected animals during both the acute and persistent phases; however, overall scores were lower than those for mice infected with L2010-2198. (Fig. 6D). Only a few mice infected with UKVDL9 displayed gait impairments and none of the mock-infected mice were scored above zero (Fig. 6C).

Listeriosis in ruminants is often referred to as circling disease due to the frequent observation of behaviors such as animals propelling themselves into corners, leaning against a stationary object, and overtly circling in the direction of the affected side of the brain (46). Severe symptoms of circling disease were noted in only a few mice, and only in the groups infected with the lineage III strain L2010-2198 or UKVDL9 (Fig. 6E). Prior to initiation of these observational studies, we also noted that one mouse fed strain UKVDL9 rapidly spun in circles when picked up by the tail; this occurred at 5 days postinfection and would have fit the criteria for a maximal deficit score. None of the mock-infected mice were given scores above zero (Fig. 6E). In general, the observed symptoms did not linger beyond the acute phase of infection (Fig. 6F).

### Mice that survive infection with neurotropic lineage III strains are protected against subsequent lethal challenge.

**FIG 7:**
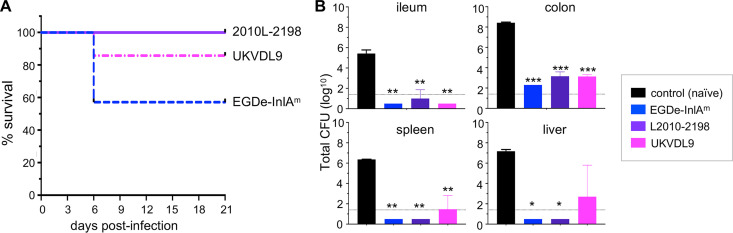
Mice that survive infection with neurotropic lineage III strains of L. monocytogenes are protected against subsequent challenge with EGDe-InlA^m^. (A) Survival curves for pooled data from neurological observations in experiment 1 (3 mice per strain) and experiment 2 (4 mice per strain). (B) Mice that recovered from experiment 1 were challenged with 1.3 × 10^10^ CFU of L. monocytogenes EGDe-InlA^m^ 5 weeks after the initial infection. Total numbers of CFU in tissue homogenates were determined 72 h later. Data were analyzed by one-way ANOVA with Dunnett’s multiple-comparison test; asterisks indicate mean values different from those for the naive control group, which was mock infected during experiment 1. *, *P < *0.5; **, *P < *0.01; ***, *P < *0.001.

Despite the extensive level of neurological impairment caused by L. monocytogenes infection, the overall mortality rate using the foodborne model was low. Combined survival data for the two neurological observation experiments are shown in Fig. 7A. No mice infected with strain L2010-2198 died during the observation period, and only 14% of mice infected with strain UKVDL9 died, with all deaths occurring by 6 days postinfection. In contrast, 43% of mice fed mouse-adapted L. monocytogenes EGDe-InlA^m^ succumbed to the infection. Taken together, these results indicated that oral transmission of neurotropic lineage III strains resulted in a model system that closely mimicked human rhombencephalitis, including severe acute neurological symptoms, persistent low-level deficits in some animals, and an overall low mortality rate in young, otherwise healthy animals.

A key feature of standard mouse models of listeriosis using lineage II reference strains such as EGDe or 10403s is the robust memory immune response that is induced during primary infection (47). To find out if oral infection with the lineage III strains generated protective immunity, the animals from experiment 1 were allowed to recover and then challenged with a lethal dose of L. monocytogenes EGDe-InlA^m^ at 5 weeks after the initial infection. As shown in Fig. 7B, all mice that received a secondary challenge had lower bacterial burdens than mice in the naive control group that was mock infected during experiment 1. Mice originally infected with strain L2010-2198 were as protected as mice that were previously infected with EGDe-InlA^m^, with no bacteria detected in the spleens and livers of either group. In the gut, the immunized mice had 100,000-fold fewer CFU in the colon and 10,000-fold fewer in the ileum. Thus, neurotropic infection did generate a memory immune response that protected the animals against secondary lethal challenge.

### Neurotropic L. monocytogenes strains can persist in the lumen of the gut but are not maintained in a gallbladder reservoir.

**FIG 8:**
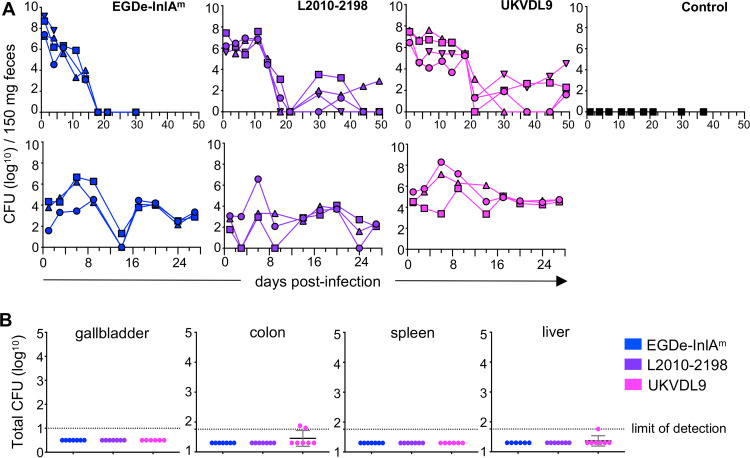
Long-term fecal shedding is not caused by a reservoir of L. monocytogenes in the gallbladder. (A) Numbers of CFU shed in feces were monitored following infection with 1 × 10^9^ CFU of the indicated L. monocytogenes strains. The top row shows data for the set of mice (experiment 2) whose results are shown in Fig. 4 and 5 with additional data points extending out to 49 dpi; the bottom row represents a separate experiment (experiment 3) not included in the neurological observation study. (B) Total numbers of CFU from whole-organ homogenate following long-term infection. Data were pooled from experiment 2 (51 dpi) and experiment 3 (31 dpi).

During experiment 2, mice infected with lineage III strain UKVDL9 were still shedding L. monocytogenes at the end of the study (Fig. 5B), so fecal monitoring was continued for all experimental groups. Surprisingly, fecal counts for both lineage III strains trended back upward in some mice during this extended time period (Fig. 8A). Since we had not done extended monitoring of fecal counts during the first trial, we then infected a third set of mice to monitor fecal shedding (experiment 3). During this experiment, long-term persistence in the gut lumen was observed for all three strains of L. monocytogenes (Fig. 8A). Hardy et al. previously showed that the gallbladder could be a reservoir for extracellular growth of L. monocytogenes; in those animals, the gastrointestinal tract was continuously reinfected during feeding as bile was excreted from the gallbladder to the small intestines (48, 49).

To determine if the neurotropic L. monocytogenes strains had established a reservoir in the murine gallbladder, we quantified the bacterial burdens in the tissues of mice used in experiments 2 and 3. As shown in Fig. 8B, no CFU were detected in the gallbladders of any mouse. The systemic infection had been cleared, as indicated by a lack of CFU in either the spleen or the liver. Two mice from the L. monocytogenes UKVDL9 group had low levels of CFU associated with the colon tissue either at or just above the limit of detection (Fig. 8B). These data suggested the continued fecal shedding of L. monocytogenes was due to establishment in the lumen of the colon and not caused by long-term colonization of the gallbladder.

## DISCUSSION

Listeriosis can present as rhombencephalitis, a focal brain stem infection, in young otherwise healthy adults who often recover from the infection, but it may have lingering neurological effects. It has been difficult to study this particular infection due to the lack of a small animal model that faithfully recapitulates key features of the human disease. The results presented here suggest that foodborne transmission in mice is a promising model system to study the pathogenic mechanisms used by neurotropic strains of L. monocytogenes to cause brain stem infection. Lineage III strain L2010-2198, originally isolated from a human patient with rhombencephalitis, preferentially infected the base of the brain and caused widespread acute and some persistent neurological symptoms in mice. Importantly, no mortality was observed in any mice infected with this strain. The data presented here support the hypothesis that any virulent L. monocytogenes that can reach high titer in the bloodstream may disseminate hematogenously during end-stage disease to cause diffuse brain infection, but only certain neurotropic strains that can directly invade the nervous system can cause nonlethal focal brain stem infection.

The mouse model we describe here has several advantages over previous model systems used to mimic rhombencephalitis in laboratory animals. In an early study, L. monocytogenes strain 511 was injected into the lips of goats, and this resulted in circling and other neurological signs; however, the infection was lethal for most of the animals (50). More recently, the Oevermann group has done elegant studies of rhombencephalitis in sheep and cows (20, 51–53), but work with large animals is expensive, and these model systems lack the extensive mutant and transgenic tools available for the study of host responses to infection in mice. Another group inoculated low doses of strain EGD into the middle ear of gerbils, causing a persistent otitis media that eventually led to brain infection (54). Similar to the work we report here, they observed neurological deficits, including ataxia and circling, starting at 5 days postinfection. However, all of the gerbils also had detectable bacteremia throughout the study, so concurrent hematogenous spread seems likely. Pagelow et al. recently described a nasal inoculation model in 1-day-old neonate mice that led to infection of the brain parenchyma and spread of L. monocytogenes along sensory neurons (55). Colonization of the brain did not occur in older (11-day-old) pups, suggesting that immune compromise was a key feature of this model, similar to a previous study that injected bacteria into the snouts of adult RAG1-deficient mice (56). Thus, the nasal inoculation route may closely model the late-stage listeriosis that occurs when neonates aspirate L. monocytogenes-contaminated amniotic fluid, but it may not be as useful for studying rhombencephalitis that occurs in otherwise healthy adults.

It is difficult to pinpoint the exact time frame in which orally acquired L. monocytogenes organisms enter the brain during either natural or experimental infections. The relatively small number of bacteria needed to cause inflammatory disease in the brain (10^3^ total CFU for strain L2010-2198) precluded our use of a bacterial luciferase imaging system, since the lower limit of detection is approximately 10^5^ CFU (49). In our previous work, L. monocytogenes organisms were not found in the brain at 3 days postinfection, but CFU were detected in most animals at 5 days (34, 37). Thus, in this study, we focused on the 5-day-postinfection time point to ensure that bacteria would be recovered from each mouse. We might have captured earlier invasion events and possibly found more animals with highly localized foci of infection in the brain stem if we had looked at time points in the 96- to 120-h-postinfection range. However, this would be a more inefficient use of animals, since the number of mice without detectable CFU in the brain would likely increase substantially.

A review of 123 human rhombencephalitis cases revealed that two thirds of the lesions identified by magnetic resonance imaging (MRI) were in either the medulla oblongata or pons of the brain stem, and the remaining third were in the cerebellum (9). Three patients in particular were highlighted who had involvement of the cerebellopontine angle, an area between the brain stem and the cerebellum that contains cranial nerves. Oevermann et al. examined the brains of naturally infected ruminants and also found that lesions were not restricted to the brain stem (42). In this study, we found preferential colonization of the brain stem and cerebellum in all infected mice, and pathological lesions were observed primarily in the brain stem. Some of the infected animals used here did not have pathological lesions in the brain. This may be due to the early time point chosen, since the lesions were more severe in a small group of mice that were examined at 7 days postinfection (data not shown). Indeed, Altimira et al. reported similar findings using strain P-14B in Swiss CD1 mice and found that the number of mice with pathological lesions increased at 11 days postinfection (25).

It has been hypothesized that L. monocytogenes organisms reach the brain stem by directly invading one of the cranial nerves that innervate the upper gastrointestinal tract (19, 57). In ruminants, who spend many hours chewing regurgitated food, this is thought to occur mainly in the oral cavity, and not surprisingly, the trigeminal (V) and facial (VII) nerves are most commonly affected (42). In humans, progressive deficits of cranial nerve function most frequently involve the trigeminal, abducens (VI), facial, glossopharyngeal (IX), and vagus (X) nerves (9, 10). The relatively larger number of cranial nerves affected in human patients suggests that either there are multiple opportunities for neuronal invasion during transmission, or that the bacteria spread to affect multiple nerve nuclei once in the brain stem. Some of the symptoms associated with rhombencephalitis are difficult to assess in mice. For example, many patients report experiencing headache, diplopia (double vision) or eye movement disorders, and numbness in particular dermatomes (6, 8, 9). Cerebellar ataxia is also common in patients (9) and was readily demonstrated here by use of the ledge test. In general, the frequency of occurrence and persistence of the neurological deficits we observed in mice were consistent with symptoms that have been reported during human cases of rhombencephalitis. The murinized variant of the lineage II strain EGDe caused much greater mortality in our model than the two lineage III strains. Although death due to L. monocytogenes encephalitis in an otherwise healthy adult has been reported (58), it is not common, so EGDe-InlA^m^ is less likely to be useful for modeling human infections in the brain.

In recent years, there has been a growing awareness that use of diverse clinical isolates rather than laboratory reference strains can lead to the identification of novel virulence factors. Lineage I strains of L. monocytogenes have been the focus of much research effort, and ST1 strains within this lineage have been associated with ruminant rhombencephalitis (29, 59). In contrast, lineage III strains of L. monocytogenes are still not well studied. Early assessments indicated a very low prevalence of lineage III strains in food products and a higher incidence among veterinary isolates (∼10%) than among human clinical isolates (∼2 to 5%) (30, 60, 61), and these observations may have decreased interest in further studies of the virulence attributes of lineage III strains. However, small collections of clinical isolates do exist (33, 41, 61), suggesting that at least some strains of lineage III L. monocytogenes are virulent in humans. Bergholz et al. showed that lineage I and lineage III strains grew faster at 37°C in high salt than lineage II strains (62). This could suggest that the two lineages share an ability to quickly adapt to the osmotic stress present in the gut lumen, making them better adapted for oral transmission in either humans or animals.

The work presented here demonstrates that neurotropic strains of L. monocytogenes are not limited to any one lineage. Although analysis of phylogenetic lineage can reveal important concepts in the evolution of a bacterial species, a deeper understanding of pathogenic mechanisms is more likely to result from studying individual phenotypes that are relevant to human disease. In support of this idea, Aguilar-Bultet et al. recently analyzed gene content and single-nucleotide variants in 225 strains of L. monocytogenes, a collection that included both clinical and environmental isolates from lineages I and II (59). They found that there was not a clear signature that defined a pathogenic versus a nonpathogenic strain based solely on phylogenetic lineage. Comparative genomic analysis of strains that share pathogenesis phenotypes, regardless of phylogenetic lineage, is likely to be a more productive way to identify novel virulence mechanisms, and the mouse model described here will be useful to assess the *in vivo* relevance of candidate virulence genes.

## MATERIALS AND METHODS

### Bacteria.

L. monocytogenes strains used in this study are listed in Table 1. Strain EGDe and the InlA^m^-expressing variant were obtained from W.-D. Schubert (38). The variant was mouse adapted by modifying two amino acids in the surface protein InlA such that a strong interaction with murine E-cadherin could occur to promote efficient invasion of the gut mucosa following oral transmission. Bacteria were cultured statically in brain heart infusion (BHI) medium (Difco) at 30°C, and aliquots were prepared and stored at −80°C until use as described previously (63).

### Mice.

Four-week-old female BALB/cByJ mice (stock number 001026) were purchased from The Jackson Laboratory (Bar Harbor, ME) and transferred to a specific-pathogen-free facility with a dark cycle from 9 a.m. to 7 p.m. Mice were given at least 2 weeks to acclimate to the reversed light cycle prior to use in infection studies between 6 and 9 weeks of age. All procedures involving mice were approved by the University of Kentucky Institutional Animal Care and Use Committee (IACUC).

### Foodborne-infection model.

Mice fasted 16 to 24 h prior to infection and were housed on raised wire flooring to prevent coprophagy, as described elsewhere (36). Frozen aliquots of L. monocytogenes were thawed, incubated statically in BHI broth at 30°C for 90 min, washed once in phosphate-buffered saline (PBS; Invitrogen number 14190), and then suspended in a 3:2 mixture of melted salted sweet cream butter (Kroger) and PBS. The bacterial suspension was then applied to 2- to 3-mm pieces of white bread (Kroger) and fed to mice early in their dark cycle as described elsewhere (35). The actual inoculum for each infection was determined by placing a piece of L. monocytogenes-contaminated bread into a microcentrifuge tube containing 1 ml of PBS, vortexing for 30 s, and then preparing serial dilutions for plating on BHI agar. For the long-term observational studies, the raised wire flooring units were removed from the cages 8 days postinfection.

### Quantification of CFU in tissue homogenates.

Organs were harvested aseptically and homogenized in sterile water using a PowerGen 1000 homogenizer at 60% power. Spleens and brains were homogenized for 30 s in a 2-ml volume; livers were homogenized for 30 s in 5 ml of sterile water. Blood was collected from the heart immediately after euthanasia (typical volume of ∼100 μl) into a microcentrifuge tube containing 50 μl of 50 mM EDTA as an anticoagulant. Gallbladders were transferred to a microcentrifuge tube containing 1 ml of sterile water, processed by cutting into small pieces using sterile scissors, and then vortexed at maximum speed for 1 min. For all organs, serial dilutions were prepared in sterile water, plated on BHI agar, and incubated at 37°C overnight. For some experiments, mice were sedated with xylazine and ketamine and cardiac perfusion was performed using 20 ml of Hanks’ balanced salt solution (Invitrogen number 14175). Perfusion efficiency was assessed by observing a blanched liver, a lack of visible veins in the ear flap, a light and glassy appearance of the eyes, no bleeding upon cutting the skull, and no visible veins in the brain (see Fig. S2).

### Multilocus sequence typing.

Chromosomal DNA isolated from each L. monocytogenes strain using the method of Flamm et al. (64) was diluted 1:10 and then used as a template for PCR to amplify seven housekeeping genes (*abcZ*, *bglA*, *cat*, *dapE*, *dat*, *ldh*, and *lhkA*) according to the procedure outlined in reference 65 using 2× Platinum PCR Supermix (Invitrogen). No amplicons were obtained for some of the strains using the standard protocol for *lhkA*, so an alternate set of primers optimized for lineage III strains was used; a list of all oligonucleotide primers used in this study is given in Table S1. PCR products were analyzed by gel electrophoresis, purified using a QIAquick cleanup kit (Qiagen), and submitted to ACGT, Inc. (Chicago, IL), for DNA sequencing using universal primers for both strands. Consensus DNA sequences were submitted to the Institut Pasteur MLST database, which assigned an allele number for each gene and ST based on each allelic profile. All strains tested in this study corresponded to existing allelic profiles in the database except for 2010L-2198.

10.1128/mSphere.00871-20.3 TABLE S1 Oligonucleotide primers used in this study. Download Table S1, PDF file, 0.03 MB. Copyright © 2020 Senay et al. 2020 Senay et al. This content is distributed under the terms of the Creative Commons Attribution 4.0 International license.

### PCR amplification of *inlF*.

Initial attempts to amplify *inlF* from the lineage III strains using primers (inlF-Forward and inlF-Reverse) described previously (66) were unsuccessful. Primers designed to amplify the region surrounding inlF (0408 FW-top and 0410 REV-bottom) were based on the genome sequence of lineage III strain M7 (GenBank accession number NC_017537.1). PCR was performed using 2× Platinum PCR Supermix, with an initial denaturation at 94°C for 2 min followed by 35 cycles of denaturation at 94°C for 30 s, annealing at 60°C for 30 s, and extension at 72°C for 2.5 min, followed by electrophoresis in a 1.2% agarose gel.

### Histopathology.

Brains were fixed in 10% neutral buffered formalin overnight and then transferred to 70% ethanol. Brains were bisected medially and embedded in paraffin. Thin sections from each half were prepared at midline, and at intervals of approximately 400 μm. Slides were stained with hematoxylin and eosin for histological analysis, and a separate Gram-stained slide was prepared to look for bacteria. For each experiment, the tissues were blinded prior to processing and then examined by a neuropathologist (F.G.G.).

The criteria used to define a pathology score were as follows: for microabscesses, 0 = none observed, 1 = single small microabscess, 2 = few small to large microabscesses, 3 = moderate number of microabscesses, possibly coalescing, 4 = high number of coalescing microabscesses involving a large area of the parenchyma; for perivascular cuffing, 0 = none observed, 1 = single layer of perivascular inflammatory cells (predominately lymphocytes), 2 = two layers of cells, 3 = three to four layers, 4 = more than four layers of cells; for ependymitis/ventriculitis, 0 = no abnormality observed, 1 = focal mild abnormality, 2 = focal severe or multifocal mild abnormality, 3 = multifocal severe abnormalities. Meningitis was scored only as absent or present and was not graded or localized, since the meninges were not uniformly intact for each brain sample. The relative amount of bacteria present was estimated using a scale from 0 to 4: 0 = no bacteria observed, 1 = single or few bacteria, 2 = moderate number of bacteria, 3 = high number of bacteria in a few foci, 4 = densely packed bacteria.

### Quantification of L. monocytogenes in the intestinal tract.

Feces were collected from each mouse using sterile forceps, placed into preweighed microcentrifuge tubes, suspended in sterile water at 150 mg/ml, mashed slightly with a sterile wooden applicator, vortexed at maximum speed for 30 s. and quick-spun to pellet the fecal matter while leaving the bacteria in the supernatant. Ilea and colons were flushed with 8 ml of cold PBS. The flushed tissues were minced with a sterile scalpel blade, transferred to a flat-bottom 10-ml centrifuge tube containing 2 ml of sterile water, and homogenized for 1 min. Serial dilutions of each sample were prepared in sterile water and plated on BHI agar supplemented with 15 g/liter lithium chloride and 10 g/liter glycine (BHI/L+G) to inhibit the growth of most intestinal microbiota, as described previously (36). Plates were incubated at 37°C for 48 h before CFU were counted; suspect colonies were confirmed using CHROMagar *Listeria* indicator plates.

### Neurological deficit observations.

Two separate trials were performed. In each experiment, blinded groups of mice were infected with a single strain of L. monocytogenes or mock infected by being given uncontaminated bread. Mice were observed twice daily by two different investigators to look for the presence of gait impairment, whisker paralysis, ptosis, or symptoms of circling disease. Cerebellar ataxia was assessed by performing a ledge test as described previously (67). The rubric used to assess these deficits and assign a score to each mouse is shown in Fig. S2.

### Statistical analysis.

All statistical analysis was performed using Prism version 8.2.0 for Mac (Graph Pad). In the figures, results for which *P* values were less than 0.5 are indicated by asterisks. The specific tests used for analysis are indicated in the figure legends.
