# Supplementary figures and images for: Neurotropic Lineage III Strains of Listeria monocytogenes Disseminate to the Brain without Reaching High Titer in the Blood
Source: mSphere. 2020 Sep 16;5(5):e00871-20. doi: 10.1128/mSphere.00871-20 (PMC7494839; doi:10.1128/mSphere.00871-20)

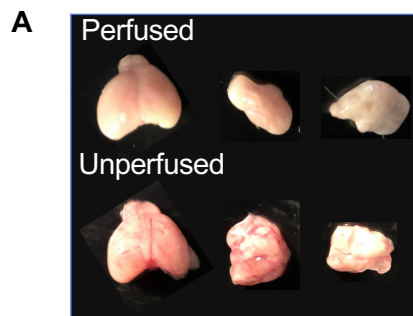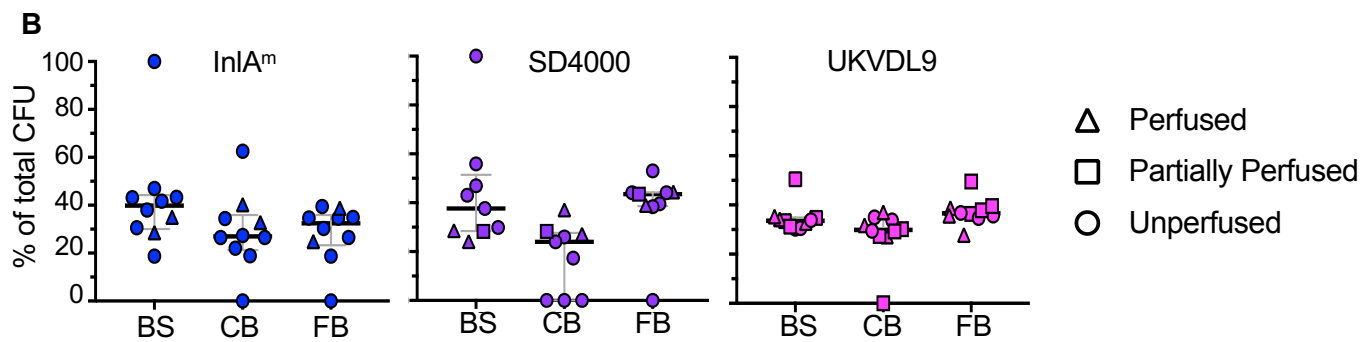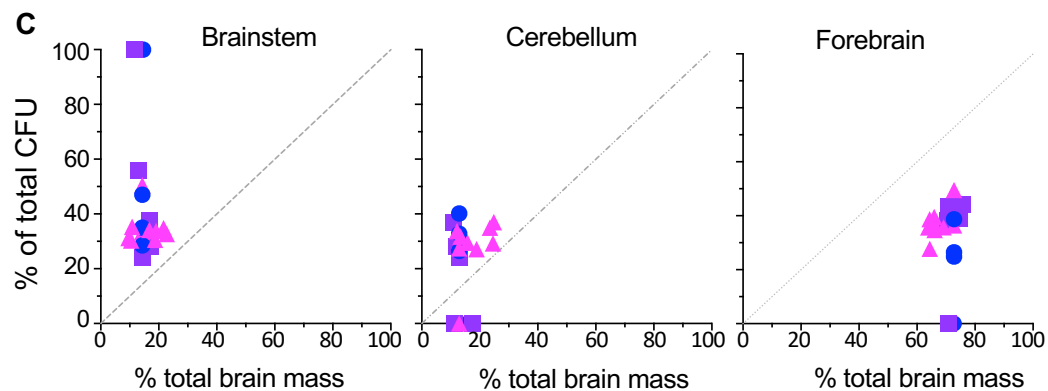

Supplement: FIG S1 [file mSphere.00871-20-sf001.pdf]
