# Supplementary material for: Neurotropic Lineage III Strains of Listeria monocytogenes Disseminate to the Brain without Reaching High Titer in the Blood
Source: mSphere. 2020 Sep 16;5(5):e00871-20. doi: 10.1128/mSphere.00871-20 (PMC7494839; doi:10.1128/mSphere.00871-20)
Supplement: FIG S2 [file mSphere.00871-20-sf002.pdf]

	0	1	2	3
**Ledge Test**	mouse walks and lowers normally	mouse slips while walking on ledge	mouse walks but will not lower into home or test cage	mouse hits head when lowering OR will not move despite prompting
**Gait**	mouse walks normally	mouse walks with an apparent limp or tremor	mouse’s abdomen drags the ground, OR has a hunched back (kyphosis)	mouse exhibits both symptoms (1 + 2)
**Circling Disease**	no symptoms noted	mouse is dependent on wall for support OR has a head tilt	mouse circles while walking in the cage or while suspended by tail	mouse exhibits both symptoms (1 + 2)
**Whisker Paralysis**	mouse has full range of typical whisker function	whiskers project backwards but have full range of motion	whiskers are “twitchy” and do not consistently move in response to an object	whiskers are immobile and do not interact with presented object
**Ptosis**	eyes are fully open	one eyelid has slight droop	Involvement of both eyes with slight droop	one or both eyes have significant closure
