# Supplementary material for: Neurotropic Lineage III Strains of Listeria monocytogenes Disseminate to the Brain without Reaching High Titer in the Blood
Source: mSphere. 2020 Sep 16;5(5):e00871-20. doi: 10.1128/mSphere.00871-20 (PMC7494839; doi:10.1128/mSphere.00871-20)
Supplement: TABLE S1 [file mSphere.00871-20-st001.pdf]

FIG S1 Oligonucleotide primers used in this study

Primer Name	Sequence*	Amplicon (bp)
FWD-Seq	5'-GTTTTCCCAGTCACGACGTTGTA-3'	n/a
REV-Seq	5'-TTGTGAGCGGATAACAATTT-3'	n/a
abcZ-forward	5'- **GTTTTCCCAGTCACGACGTTGTA**TCGCTGCCACTTTTATCCA -3'	650
abcZ-reverse	5'- **TTGTGAGCGGATAACAATTT**CTCAAGGTCGCCGTTTAGAG -3'	
bglA-forward	5'- **GTTTTCCCAGTCACGACGTTGTA**GCCGACTTTTTATGGGGTGGAG -3'	501
bglA-reverse	5'- **TTGTGAGCGGATAACAATTT**CCGATTAAATACGGTGCGGACATA -3'	
cat-forward	5'- **GTTTTCCCAGTCACGACGTTGTA**ATTGGCGCATTTTGATAGAGA -3'	599
cat-reverse	5'- **TTGTGAGCGGATAACAATTT**CAGATTGACGATTCCTGCTTTTG -3'	
dapE-forward	5'- **GTTTTCCCAGTCACGACGTTGTA**CGACTAATGGGCATGAAGAACAAG -3'	565
dapE-reverse	5'- **TTGTGAGCGGATAACAATTT**CATCGAACTATGGGCATTTTTACC -3'	
dat-forward	5'- **GTTTTCCCAGTCACGACGTTGTA**GAAAGAGAAGATGCCACAGTTGA -3'	611
dat-reverse	5'- **TTGTGAGCGGATAACAATTT**CTGCGTCCATAATACACCATCTTT -3'	
ldh-forward	5'- **GTTTTCCCAGTCACGACGTTGTA**GTATGATTGACATAGATAAAGA -3'	744
ldh-reverse	5'- **TTGTGAGCGGATAACAATTT**CTATAAATGTCGTTCATACCAT -3'	
lhkA-F3	5'- **GTTTTCCCAGTCACGACGTTGTA**GCAAGTTTTGAATACGTATCAGCG -3'	570
lhkA-R2	5'- **TTGTGAGCGGATAACAATTT**CTACGCATTTCATGAGAAACATCAG -3'	
inlF-Forward	5'- AGAAGCGGAAATTTGCATATTAA -3'	*Lm* EGD-e: 1603<br>Lin. III *Lm*: 0
inlF-Reverse	5'- ACCAACCATCAAATGTATAACCTTG -3'	
0408 FW-top	5'- CCGGTGACACCATTGTTTGTTTACC -3'	*Lm* EGD-e: 3,011<br>Lin. III *Lm*: 341
0410 REV-bottom	5'- TAA AGG GCT TGT GAC CGA GGT TGG -3'	

*Bolded regions denote the forward and reverse sequencing primers
